# Analysis of Autophagy‐Related Gene Signature Associated With Clinical Prognosis and Immune Microenvironment in Colorectal Cancer

**DOI:** 10.1155/mi/3900151

**Published:** 2026-03-28

**Authors:** Dazhuang Miao, Yushui Song, Liang Zhou, Guanying Liang, Yan Wang, Wei He, Luyu Huang, Hongnan Lu, Shixiong Jiang, Yunhe Jia, Zhiwei Li, Jinxue Tong

**Affiliations:** ^1^ Department of Colorectal Cancer Surgical Ward 2, Harbin Medical University Cancer Hospital, Harbin, China, hrbmu.edu.cn; ^2^ Department of Gastroenterology Ward 1, Harbin Medical University Cancer Hospital, Harbin, China, hrbmu.edu.cn; ^3^ Department of Colorectal Cancer Surgical Ward, Cancer Hospital of Chinese Academy of Medical Sciences, Beijing, China; ^4^ Department of Pathology, Harbin Medical University Cancer Hospital, Harbin, China, hrbmu.edu.cn; ^5^ Department of General Surgery, Zigong Third People’s Hospital, Zigong, China, www.zgsyy.cn

**Keywords:** autophagy, colorectal cancer, genes, immunotherapy, prognosis

## Abstract

**Background:**

Autophagy has a critical involvement in the initiation and progression of various cancers, including colorectal cancer (CRC). The feasibility of using autophagy‐related genes (ATGs) as prognostic tools for CRC patients is yet to be determined.

**Methods:**

RNA sequencing data and clinical information for CRC were obtained from The Cancer Genome Atlas (TCGA) (training set) and Gene Expression Omnibus (GEO) datasets GSE39582 and GSE44076 (validation). ARGs were retrieved from the Human Autophagy Database, and differentially expressed ARGs (DAGs) were identified using the “limma” R package. Prognostic signature DAGs were established via univariate Cox and LASSO Cox regression analyses. The obtained signature was validated through expression analysis, autophagy scoring, survival prediction, and correlation with immune status. Immunohistochemistry assays and in vitro functional experiments in SW480 cells were performed to assess the biological roles of selected DAGs, particularly WIPI2.

**Results:**

We constructed an 11‐gene prognostic signature (CANX, NRG1, WIPI1, EIF2AK3, WDR45, PELP1, ULK1, WIPI2, DAPK1, ULK3, and MAP1LC3C), with high‐risk patients showing significantly reduced overall survival compared to low‐risk patients. WIPI2, highly expressed in SW480 cells, was selected for functional validation. Knockdown of WIPI2 inhibited cell proliferation, suppressed autophagy (decreased LC3B‐II, increased p62), and promoted apoptosis (increased cleaved caspase‐3). These findings confirm the pro‐survival role of WIPI2 in CRC. The prognostic signature remained independently predictive after adjusting for clinical factors and showed a strong correlation with immune infiltration in TCGA CRC samples.

**Conclusion:**

The autophagy‐related signature independently predicts CRC prognosis and guides immunotherapy strategies.

## 1. Introduction

Colorectal cancer (CRC), comprising malignancies in the colon, anal canal, and rectum, ranks as the fourth most prevalent cancer globally and is the third leading cause of cancer‐related fatalities. It was responsible for ~1.9 million new cases and 900,000 deaths in 2020 [1, 2]. The primary treatment modalities for CRC include surgical intervention, chemotherapy, targeted therapy, and immunotherapy [3]. Notably, surgery is typically an option for patients diagnosed in the early stages of CRC, whereas those with advanced disease often exhibit resistance to chemotherapy and targeted therapies [4]. This underscores the necessity of advancing our understanding of CRC’s molecular pathogenesis, aiming to enhance early detection and treatment methodologies.

Recent advancements in research have uncovered that autophagy proteins hold significant prognostic value in CRC [5]. Autophagy, a universally conserved process in eukaryotic cells, plays a crucial role in degrading and recycling macromolecules, thereby replenishing essential substances for normal cellular functions [6]. This process is particularly active under stress conditions like organelle damage, abnormal protein production, and nutrient scarcity. It involves the formation of autophagosomes that encapsulate the targeted components and fuse with lysosomes to form autophagolysosomes, initiating degradation and recycling [7]. The influence of autophagy on cellular health is complex; it can either support cell survival or lead to growth inhibition and apoptosis [8]. Various autophagy‐related genes (ATGs) intricately regulate autophagy, influencing CRC in multiple ways. For example, the downregulation of PLK4‐induced autophagy can trigger tumor dormancy, while inhibiting autophagy results in apoptosis of CRC dormant cells [9]. PCK1 impedes CRC growth by deactivating UBAP2L phosphorylation at serine 454 and enhancing autophagy [10]. However, while existing studies have explored the role of autophagy in CRC progression and patient response to treatment, focusing on specific ARGs [11], a comprehensive analysis of how these ARG expressions predict CRC patient prognoses remains limited.

Consequently, to enhance the prognostic assessment of CRC patients, this study embarked on identifying prognostically significant ARGs through analysis of The Cancer Genome Atlas (TCGA) and Gene Expression Omnibus (GEO) datasets. Subsequently, we established an ATG signature, which holds promise for guiding the development of innovative therapeutic strategies in CRC management.

## 2. Methods

### 2.1. Acquirement of Target Datasets

**Figure 1:**
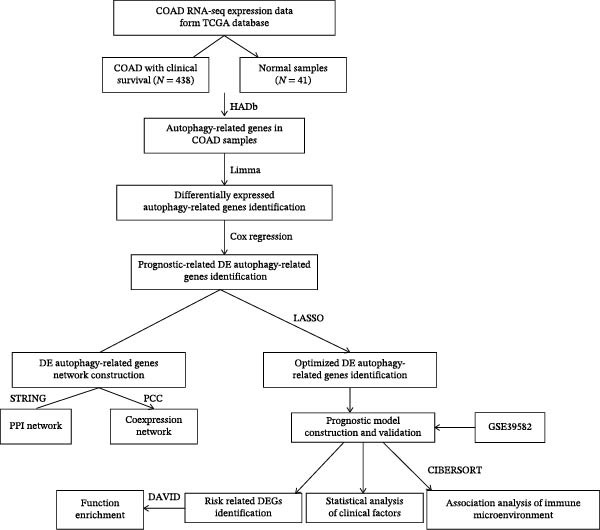
Flowchart indicating the study design of the present work.

The gene expression data, standardized using log_2_ (FPKM + 1), along with associated clinical information for 438 CRC patients and 41 control subjects, were obtained from the TCGA database (accessed on 11 May 2022) and used for the training cohorts. For the validation set, the dataset (GSE39582) on 519 CRC patients and the dataset (GSE44076) on 246 patients from the GEO databases were utilized. The overall study design and the different samples that were included at every stage of the analysis were illustrated as a flowchart in Figure 1.

### 2.2. Screening of Prognosis‐Related Differentially Expressed ARGs (DAGs)

ARGs were sourced from the Human Autophagy Database (http://www.autophagy.lu/) [12]. We applied an intergroup *t*‐test with a false discovery rate (FDR) < 0.05 and |log_2_FC| > 0.5 as the screening criterion to discern DAGs between normal and tumor samples in the TCGA CRC dataset. Furthermore, hierarchical clustering heat maps depicting the levels of gene expression were generated using the pheatmap package [13]. Employing the “survival package (version 2.41)” [14], we conducted a univariate Cox regression analysis to screen prognosis‐related DAGs. A log‐rank *p*‐value < 0.05 was set as the threshold for identifying significant correlations. Additionally, the Kaplan–Meier (KM) method was utilized to assess the differences in survival outcomes.

### 2.3. Network Construction of Prognosis‐Related DAGs

Within the STRING (Version 11.0, http://string-db.org/) database [15], we searched for interactions among the protein products of the screened prognosis‐related DAGs. An interaction score threshold of greater than 0.4 was established as the criterion for selecting meaningful interactions, which were then used to construct an interaction network. Additionally, we employed the corollary function (http://77.66.12.57/R-help/cor.test.html) to calculate the Pearson correlation coefficient (PCC) between the expression levels of these prognosis‐related DAGs in TCGA CRC samples. Pairs of relations with a *p*‐value of less than 0.05, and an absolute PCC value greater than 0.3 were deemed to have a significant correlation. These pairs were then used to construct a coexpression network. Both the interaction and coexpression networks were visually depicted using Cytoscape (Version 3.6.1, http://www.cytoscape.org/) [16].

### 2.4. Construction and Validation of Autophagy‐Risk Signature

To construct and validate an autophagy‐risk signature, we first applied the LASSO Cox regression algorithm (R package lars Version 1.2) [17] to refine prognosis‐related DAGs from the TCGA dataset, selecting the optimal penalty parameter (*λ*) through 1000 iterations of 10‐fold cross‐validation to minimize overfitting. The obtained optimal prognostic DAGs were used to calculate a risk score (RS) as follows:

RS=∑Coefgenes×Expgenes,

where “Coef_genes_” represents LASSO‐derived coefficients and “Exp_genes_” denotes log_2_‐transformed expression values from the TCGA and GSE39582 datasets. Differential expression trends of these DAGs between tumor and control samples were analyzed in TCGA and GSE44076 datasets using moderated *t*‐tests (R package limma, v3.56.0) with thresholds of |log_2_FC| > 1.0 and adjusted *p* < 0.05 (Benjamini–Hochberg correction). Diagnostic performance was assessed using receiver operating characteristic (ROC) curves (R package pROC, v1.18.5) with 2000 bootstrap replicates for AUC calculation. Autophagy scores were computed via single‐sample gene set variation analysis (ssGSEA; R package GSVA, v1.36.3) and validated through principal component analysis (PCA) and ROC analysis [18] in GSE44076. Survival differences between high/low‐risk groups (stratified by median RS) were evaluated using KM analysis (R package survival, v2.41‐1) and time‐dependent ROC curves (R package survival ROC, v1.0.3), achieving AUCs of TCGA and GSE39582 for 1–5‐year survival predictions [14].

### 2.5. Correlation Analysis of Risk Signature and Clinicopathological Characteristics

Utilizing our developed risk signature, we analyzed the distribution of clinical factors among different risk groups within the TCGA CRC samples. We then compared the clinical information of various sample subtypes using the intergroup Fisher test. To visually represent this data, hierarchical clustering heat maps were created to show the expression levels of the optimal prognostic signature DAGs along with significant clinicopathological characteristics. These heat maps were generated using the “pheatmap package” [13].

### 2.6. Functional and Pathway Enrichment Analyses

Utilizing our developed risk signature, we employed the “limma package (Version 3.34.7, available at https://bioconductor.org/packages/release/bioc/html/limma.html)” [19] to identify significantly differentially expressed genes (SDGs) between these high‐ and low‐risk groups. The criteria for significant differential expression were set as FDR < 0.05 and |log_2_FC| > 0.5. Post identification of these SDGs, we conducted Gene Ontology (GO) function and Kyoto Encyclopedia of Genes and Genomes (KEGG) pathway enrichment analyses using DAVID (Version 6.8, The Database for Annotation, Visualization, and Integrated Discovery, available at https://david.ncifcrf.gov/) [20, 21]. For this analysis, FDR < 0.05 was adopted as the threshold for identifying significant correlations.

### 2.7. Immune Infiltration Analysis

The CIBERSORT algorithm [22] was utilized to evaluate the variations in tumor‐infiltrating immune cells (TIICs) between the high‐ and low‐risk subgroups. Following this assessment, the corollary function was employed to determine the correlation between the expression levels of the optimal prognostic signature DAGs or RS and the distribution of those immune cells that showed significant differences between the subgroups.

### 2.8. Tissue Microarray

A tissue microarray comprising 99 CRC tumor specimens and 64 adjacent nontumor specimens was acquired from Harbin Medical University Cancer Hospital. None of the patients from whom these samples were taken had undergone radiotherapy or chemotherapy prior to their lung resection surgery. The CRC samples were specifically harvested from the periphery of the tumor lesions, and the pathological diagnoses were verified by at least two experienced pathologists. The collection of these tissue samples was conducted with the approval of the ethics committee at Harbin Medical University Cancer Hospital.

### 2.9. Immunohistochemistry

The paraffin‐embedded tissue samples were sectioned into 4 μm slices. These sections were then subjected to deparaffinization and antigen retrieval, which involved boiling them in a 10 mM citrate buffer solution with a pH of 6.0. To inhibit endogenous peroxidase activity, a 3% hydrogen peroxide (H_2_O_2_) solution was applied. For the primary antibodies, ULK1, WIPI2, NRG1, PELP1, DAPK1, and MAP1LC3C were used. The assessment of positivity and intensity of these stains was conducted under a light microscope by two independent pathologists, who performed their evaluations in a blinded fashion to ensure unbiased results.

### 2.10. In Vitro Cell Culture and Functional Assays

Human normal colonic epithelial cell line FHC and human CRC cell lines HT29, SW480, and HCT116 were obtained from the American Type Culture Collection (ATCC, Manassas, VA, USA). FHC cells were cultured in Dulbecco’s Modified Eagle Medium (DMEM)/F12 medium supplemented with 10% fetal bovine serum (FBS), while HT29, SW480, and HCT116 cells were maintained in DMEM containing 10% FBS and 1% penicillin–streptomycin. All cells were cultured at 37°C in a humidified incubator with 5% CO_2_. WIPI2 expression levels were examined in all four cell lines, and SW480 cells were selected for subsequent functional experiments.

Small interfering RNAs targeting WIPI2 (si‐WIPI2#1, si‐WIPI2#2, and si‐WIPI2#3) and a negative control siRNA (si‐NC) were synthesized by GenePharma (Shanghai, China). SW480 cells were transfected with siRNAs using Lipofectamine RNAiMAX (Invitrogen, USA) according to the manufacturer’s instructions, and cells were harvested 48 h after transfection to assess knockdown efficiency.

Cell viability was evaluated using the cell counting kit‐8 (CCK‐8) assay. Transfected SW480 cells were seeded into 96‐well plates at a density of 2–3 × 10^3^ cells/well, and absorbance at 450 nm was measured after incubation with CCK‐8 solution. Cell proliferation was further assessed using an EdU incorporation assay. Briefly, transfected cells were incubated with EdU reagent for 2 h, fixed with 4% paraformaldehyde, permeabilized with 0.5% Triton X‐100, and stained with fluorescent dye, followed by nuclear counterstaining with Hoechst 33342.

Autophagic flux was analyzed using a tandem mCherry–GFP–LC3B adenoviral reporter. Transfected SW480 cells were seeded into confocal dishes and transduced with adv‐mCherry‐LC3B‐GFP at a multiplicity of infection (MOI) of 50. After 48 h, cells were incubated with Earle’s balanced salt solution (EBSS; Beyotime, C0213) for 10 h to induce autophagy. Fluorescent images were acquired using a confocal laser scanning microscope, in which yellow puncta (GFP^+^/mCherry^+^) indicated autophagosomes and red‐only puncta (GFP^−^/mCherry^+^) represented autolysosomes.

Total RNA was extracted using TRIzol reagent (Invitrogen, USA) and reverse‐transcribed into cDNA. Quantitative real‐time PCR was performed using SYBR Green Master Mix, and WIPI2 mRNA expression was quantified using GAPDH as an internal control with the 2^-ΔΔCt^ method. For protein analysis, total proteins were extracted using RIPA lysis buffer and subjected to Western blotting with primary antibodies against WIPI2 (Abcam, Cambridge, UK; ab105459), LC3B (Cell Signaling Technology, Danvers, MA, USA; #3868), p62 (Cell Signaling Technology; #5114), cleaved caspase‐3 (Cell Signaling Technology; #9661), and GAPDH (Cell Signaling Technology; #5174). Protein bands were visualized using enhanced chemiluminescence, with GAPDH serving as a loading control.

### 2.11. Statistical Analysis

All graphical representations and statistical analyses in our study were carried out using R software (version 3.6.1), provided by the R Foundation for Statistical Computing. We employed two‐tailed tests for our analyses, setting a *p*‐value or FDR of less than 0.05 as the threshold for deeming results statistically significant.

## 3. Results

### 3.1. Identification of Prognosis‐Related DAGs

To elucidate the molecular underpinnings of autophagy in CRC, we retrieved 232 ARGs from the Human Autophagy Database and analyzed their expression in the TCGA CRC cohort. Differential expression analysis (FDR < 0.05, |log2FC| > 0.5) identified 170 DAGs between tumor and normal tissues (Supporting Information 1: Figure S1, Supporting Information 2: Table S1). Univariate Cox regression of these DAGs revealed 25 prognosis‐associated DAGs (Table 1). To prioritize genes for survival analysis, we ranked these DAGs not only by log‐rank *p*‐values but also by effect size (hazard ratio, HR) and biological relevance based on prior evidence linking them to autophagy or CRC progression (e.g., ULK1 and MAP1LC3C regulate autophagosome formation; DAPK1 is a tumor suppressor). After adjusting for multiple testing (FDR < 0.1), ULK1, MAP1LC3C, CDKN2A, DAPK1, and ULK3 emerged as the top candidates, exhibiting both strong statistical significance (adjusted *p* < 0.05) and clinically meaningful HRs (>2.0 or <0.5). KM analysis confirmed that higher expression of ULK1, CDKN2A, DAPK1, and ULK3 and lower MAP1LC3C expression correlated with significantly worse overall survival (log‐rank *p*  < 0.05; Figure 2A). Differential expression of these genes in tumor versus normal tissues was further validated (Figure 2B). While the remaining 20 DAGs were analyzed (Supporting Information 3: Table S2), their associations with survival were weaker (adjusted *p* > 0.1 or |HR| < 1.5), justifying their exclusion from subsequent mechanistic studies.

Figure 2Identification of prognosis‐related DAGs. (A) The KM curves of top five prognosis‐related DAGs. Blue and red curves represent low‐ and high‐expressed groups. (B) The expression levels of top five prognosis‐related DAGs between tumor and normal tissues in the TCGA CRC dataset. CRC, colorectal cancer; DAGs, differentially expressed autophagy‐related genes.

**Figure:**
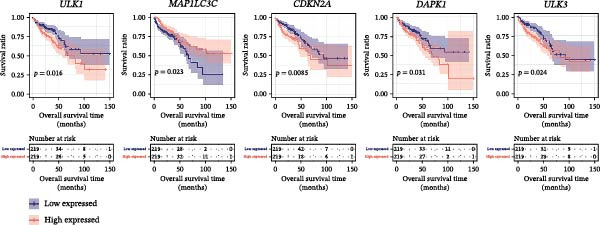
(A)

**Figure:**
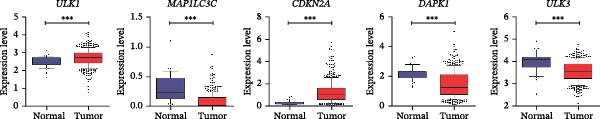
(B)

**Table 1:** A total of 25 autophagy‐related genes identified in TCGA dataset.

Symbol	Coefficient	exp (coef)	se (coef)	*z*
ULK1	0.744	2.1	0.23	3.24
MAP1LC3C	2.01	7.47	0.668	3.01
CDKN2A	0.238	1.27	0.0843	2.82
DAPK1	0.291	1.34	0.104	2.78
ULK3	0.6	1.82	0.224	2.68
ATG4B	0.634	1.89	0.239	2.65
CTSD	0.401	1.49	0.164	2.45
WIPI2	0.679	1.97	0.285	2.38
CANX	−0.393	0.675	0.168	−2.34
CHMP2B	−0.356	0.701	0.162	−2.19
ATG4C	−0.39	0.677	0.193	−2.02
BIRC5	−0.3	0.741	0.155	−1.93
WIPI1	−0.306	0.736	0.16	−1.91
NAMPT	−0.239	0.788	0.128	−1.87
SPHK1	0.181	1.2	0.098	1.84
WDR45	0.431	1.54	0.236	1.83
HSPA8	−0.294	0.746	0.169	−1.74
ZFYVE1	0.442	1.56	0.255	1.73
NRG1	−0.87	0.419	0.505	−1.72
PELP1	0.321	1.38	0.188	1.71
VEGFA	0.266	1.3	0.156	1.7
SH3GLB1	−0.355	0.701	0.209	−1.7
CAPN10	0.43	1.54	0.254	1.69
EIF2AK3	−0.374	0.688	0.222	−1.68
VAMP3	−0.324	0.723	0.193	−1.67

### 3.2. Network Construction of Prognosis‐Related DAGs

Utilizing the 25 prognosis‐related DAGs, we identified 141 protein–level interaction pairs with an interaction score exceeding 0.4. These pairs were used to construct a protein interaction network, which was visualized using Cytoscape Version 3.6.1 (Figure 3A). In parallel, we also discovered 138 relation pairs characterized by a *p*‐value below 0.05, and an absolute PCC value above 0.3. These pairs were instrumental in constructing a coexpression network, also visualized using Cytoscape Version 3.6.1 (Figure 3B).

Figure 3Network construction of prognosis‐related DAGs. The interaction (A) and coexpression networks (B) of prognosis‐related DAGs using Cytoscape Version 3.6.1. The color of each node reflects the extent of gene expression variation in COAD tumor samples, with red indicating upregulation and blue indicating downregulation. The size of the nodes denotes the significance of the correlation with survival and prognosis, where a larger node size signifies greater correlation significance. Additionally, the type of connections between nodes is also meaningful: solid connections represent significant positive correlations, while dashed connections indicate significant negative correlations.

**Figure:**
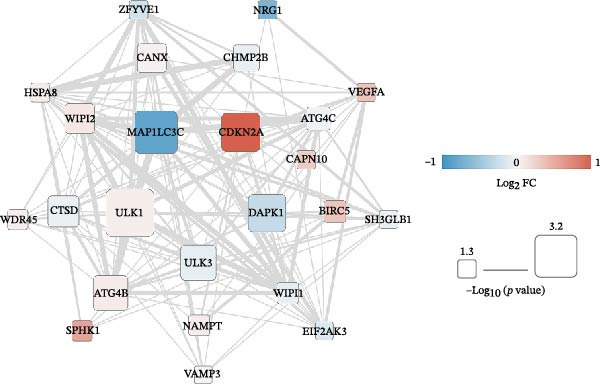
(A)

**Figure:**
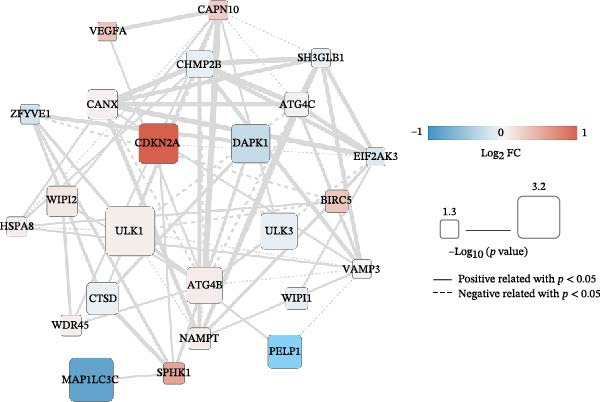
(B)

### 3.3. Construction and Validation of Autophagy‐Risk Signature

LASSO Cox regression analysis of 25 prognosis‐related DAGs identified 11 optimal genes (CANX, NRG1, WIPI1, EIF2AK3, WDR45, PELP1, ULK1, WIPI2, DAPK1, ULK3, and MAP1LC3C) via 1000 iterations of cross‐validation (Supporting Information 1: Figure S2A,B). Validation across TCGA and GSE44076 datasets using moderated *t*‐tests (limma package; |log_2_FC| > 1, Benjamini–Hochberg‐adjusted *p* < 0.05) confirmed consistent expression trends for all 11 DAGs between tumor and controls (Supporting Information 1: Figure S3; Supporting Information 4: Table S3), with 10 genes in GSE44076 showing significant differential expression (*p* < 0.05) and high diagnostic accuracy (AUC > 0.85). Autophagy scores calculated via ssGSEA (GSVA package) stratified samples into high/low groups (median threshold), with the distribution of autophagy scores across samples visualized in Figure 4A, validated by PCA clustering (Figure 4B) and cross‐dataset consistency (TCGA/GSE44076 tumor scores elevated, *p* < 0.001; Figure 4E–H). Figure 5A,B displayed the respective RS value distributions and survival time distributions for the TCGA and GSE39582 datasets. Prognostic risk stratification (median RS) revealed significantly prolonged survival in low‐risk patients (log‐rank *p* < 0.001, HR = 0.41; Figure 5C), with the 11‐gene model achieving robust predictive accuracy (TCGA: 1–5‐year AUC = 0.936–0.938; GSE39582: 0.808–0.830; Figure 5D).

Figure 4Assessment and validation of autophagy score in optimal prognostic signature DAGs. (A) Autophagy score distribution from low to high; (B) sample PCA analysis results based on autophagy score. (C) After sequencing the samples from low to high according to autophagy score, 11 gene expression levels were clustered heat maps. (D) The expression levels of 11 genes were distributed among different autophagy score groups. (E,F) Distribution of autophagy score in different sample groups in the TCGA and GSE44076 datasets. (G,H) ROC curve of sample risk based on autophagy score.

**Figure:**
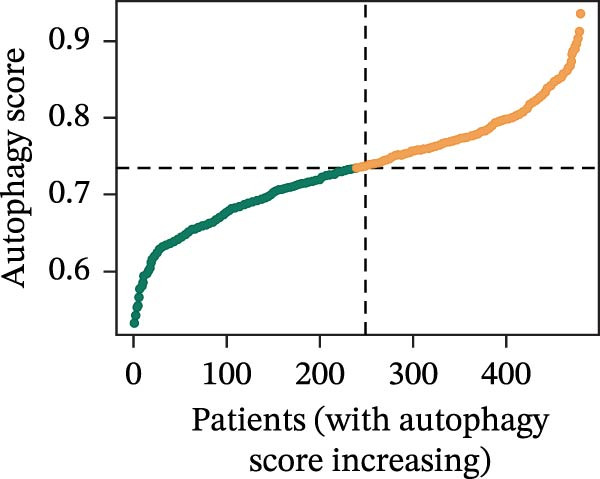
(A)

**Figure:**
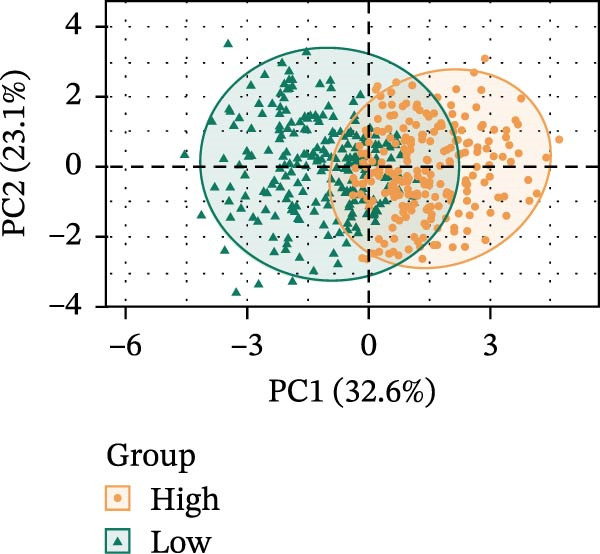
(B)

**Figure:**
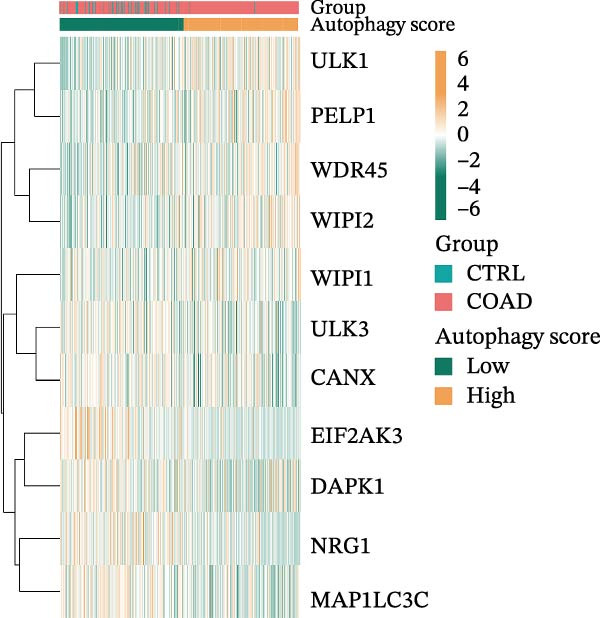
(C)

**Figure:**
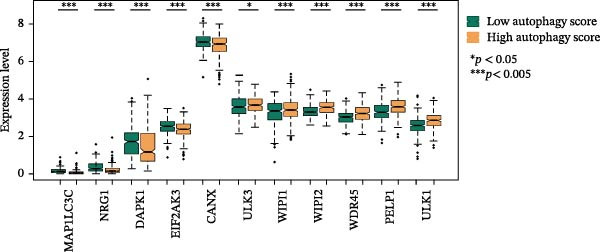
(D)

**Figure:**
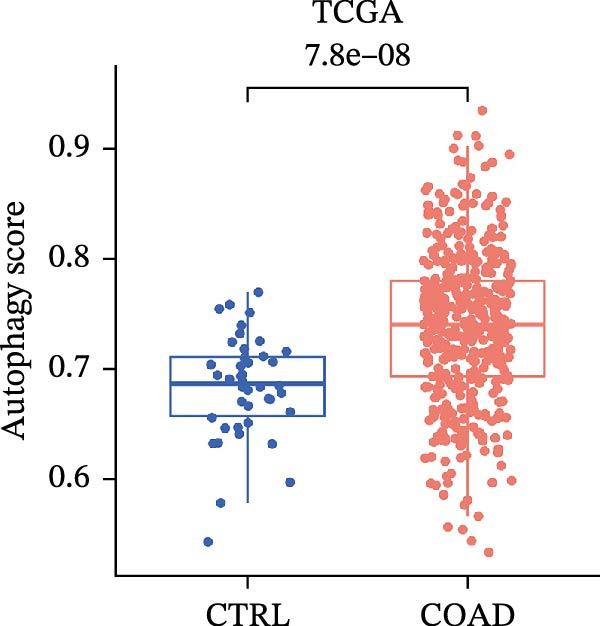
(E)

**Figure:**
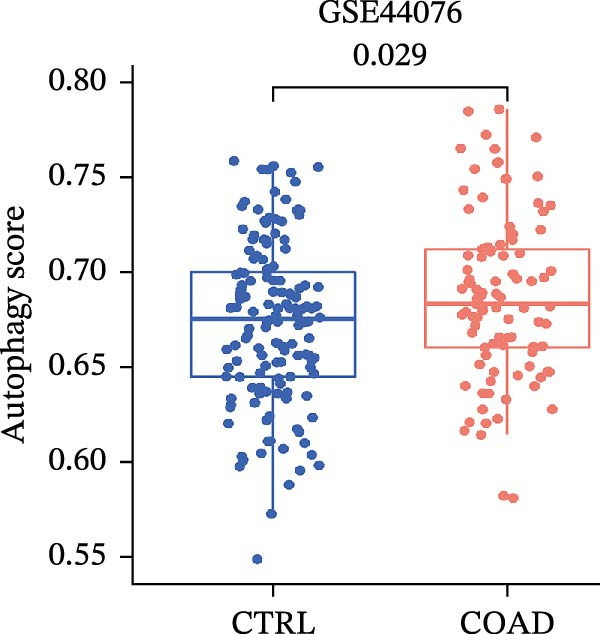
(F)

**Figure:**
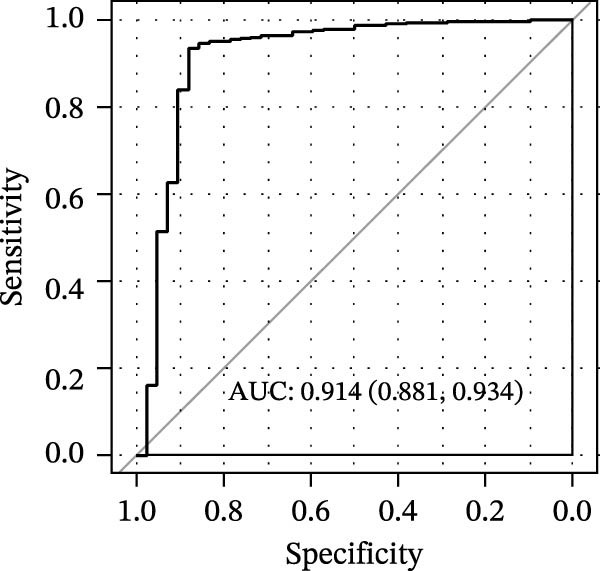
(G)

**Figure:**
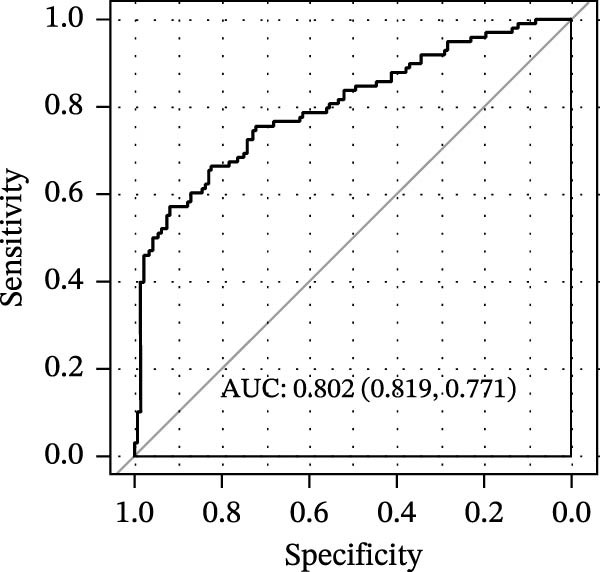
(H)

Figure 5Construction and validation of autophagy‐risk signature. The RS value distributions (A) and survival time distributions (B) of the TCGA and GSE39582 datasets were shown. (C) The Kaplan–Meier curves of the survival of patients from the high‐risk group and low‐risk group in the TCGA and GSE39582 datasets. Log‐rank *p* < 0.05 was considered statistical significance. (D) The ROC analysis and AUC value of the ROC curves for 1‐, 3‐, and 5‐year survival predictions in the TCGA and GSE39582 datasets. RS, risk score; ROC, receiver operating characteristic; AUC, area under the curve.

**Figure:**
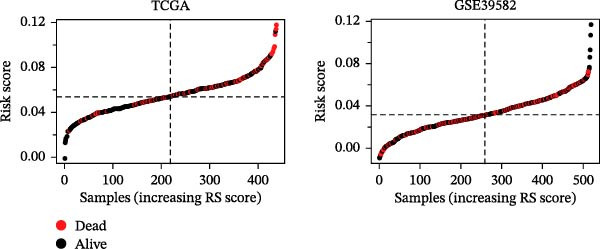
(A)

**Figure:**
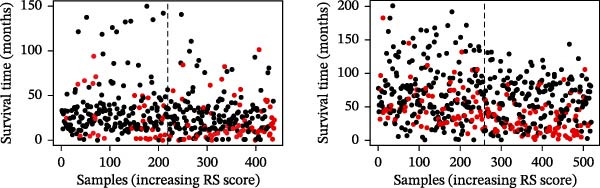
(B)

**Figure:**
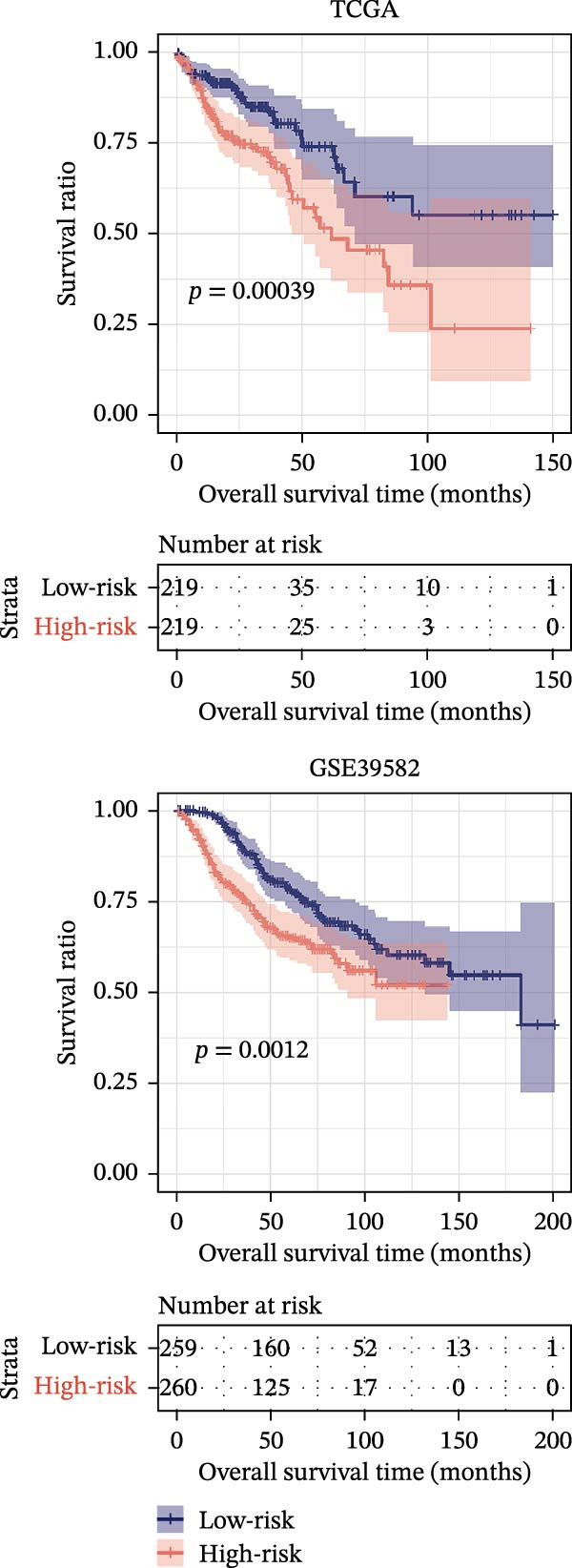
(C)

**Figure:**
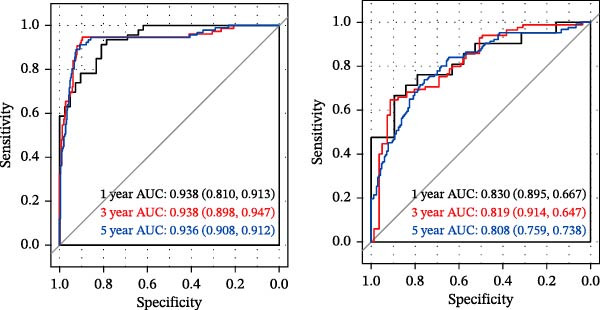
(D)

### 3.4. Correlation Analysis of Risk Signature and Clinicopathological Characteristics

**Figure 6:**
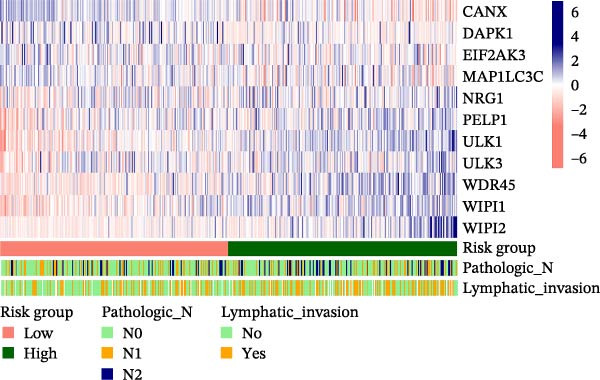
Correlation analysis of risk signature and clinicopathological characteristics. Heatmap of 11 optimal prognostic signature DAGs, alongside the distribution of pathologic N and lymphatic invasion in the TCGA CRC dataset.

A total of 438 patients from the TCGA CRC dataset, complete with clinical information such as age, gender, and clinical stage, were selected for in‐depth analysis. The intergroup Fisher test results indicated significant differences in the distribution of Pathologic N and Lymphatic invasion factors between the high‐risk and low‐risk groups (Table 2). Additionally, a heatmap was created to visualize the expression levels of the 11 optimal prognostic signature DAGs, alongside the distribution of Pathologic N and Lymphatic invasion in these TCGA CRC samples, as illustrated in Figure 6.

**Table 2:** Correlation analysis of risk signature and clinicopathological characteristics.

Characteristics total cases	Case (*n* = 438)	Group		*p*‐Value
		Low‐risk (*N* = 219)	High‐risk (*N* = 219)	
Age (years)				
≤60	134	63	71	4.68E‐01
>60	304	156	148	
Gender				
Male	233	121	112	4.44E‐01
Female	205	98	107	
Pathologic M				
M0	325	162	163	8.88E‐01
M1	60	31	29	
Pathologic N				
N0	257	132	125	4.44E‐02
N1	103	57	46	
N2	78	30	48	
Pathologic T				
T1	11	4	7	1.01E‐01
T2	76	41	35	
T3	300	156	144	
T4	51	18	33	
Pathologic stage				
I	74	36	38	7.52E‐01
II	168	89	79	
III	126	59	67	
IV	60	31	29	
History of colon polyps				
Yes	130	62	68	2.78E‐01
No	242	130	112	
Lymphatic invasion				
Yes	154	63	91	3.87E‐03
No	241	135	106	
Recurrence				
Yes	78	37	41	4.45E‐01
No	293	155	138	

### 3.5. Functional and Pathway Enrichment Analyses

Next, we identified 1320 SDGs between the high‐risk and low‐risk groups in TCGA CRC samples (Supporting Information 5: Table S4). To understand the functional implications of these SDGs, we conducted GO and KEGG analyses. These analyses revealed significant enrichment of the SDGs in 31 GO biological process categories and 10 KEGG pathways, all meeting the criterion of FDR < 0.05 (Supporting Information 6: Table S5). Notably, the top 10 enriched GO terms, which included functions such as calcium ion binding and cell adhesion, and KEGG pathways, such as neuroactive ligand–receptor interaction and calcium signaling pathway, were prominently featured, and presented in Figure 7A–B.

Figure 7Functional and pathway enrichment analyses. The top 10 GO (A) and KEGG enrichment items (B) of 1320 SDGs between the high‐risk and low‐risk groups in TCGA CRC samples. SDGs, significantly differentially expressed genes.

**Figure:**
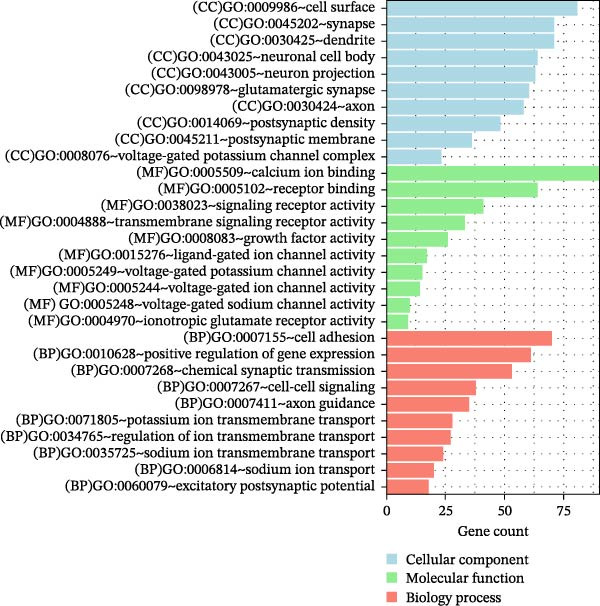
(A)

**Figure:**
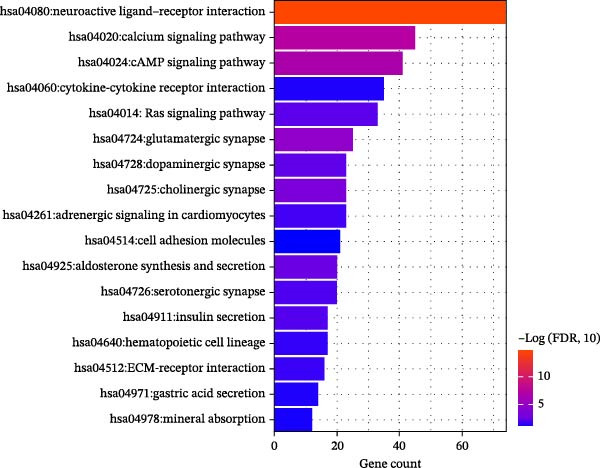
(B)

### 3.6. Analysis of Immune Status Between Low‐ and High‐Risk Subgroups

To explore the potential link between the 11 optimal prognostic signature DAGs and tumor immune response, we analyzed the correlation between the RS and 22 types of TIICs in CRC using the CIBERSORT algorithm, with the results detailed in Supporting Information 7: Table S6. Our analysis indicated a pronounced presence of T cell CD8+, T cell regulatory (Tregs), and mast cells resting in the high‐risk group. In contrast, the low‐risk group showed significant enrichment in B cell plasma, T cell CD4+ memory activated, T cell gamma delta, myeloid dendritic cell activated, mast cell resting, and Eosinophil, as depicted in Figure 8A. Additionally, Figure 8B presented a heatmap that visually captures the strong positive and negative correlations between the expression levels of these 11 optimal prognostic signature DAGs or RS and the distribution of tumor‐infiltrating cells. These insights collectively suggest a potential impact of the prognostic signature DAGs on the effectiveness of immunotherapeutic approaches in CRC patients.

Figure 8Relationship between the prognostic autophagy‐risk signature and immune responses in CRC. (A) The boxplot displayed the different proportions of tumor‐infiltrating cells between the two risk groups (blue was the low‐risk group and red was the high‐risk group). (B) Heat map showing the correlation between 11 optimal prognostic signature DAGs or RS and significantly different proportions of tumor‐infiltrating cells.  ^∗^ *p* < 0.05,  ^∗∗^ *p* < 0.01,  ^∗∗∗^ *p* < 0.001.

**Figure:**
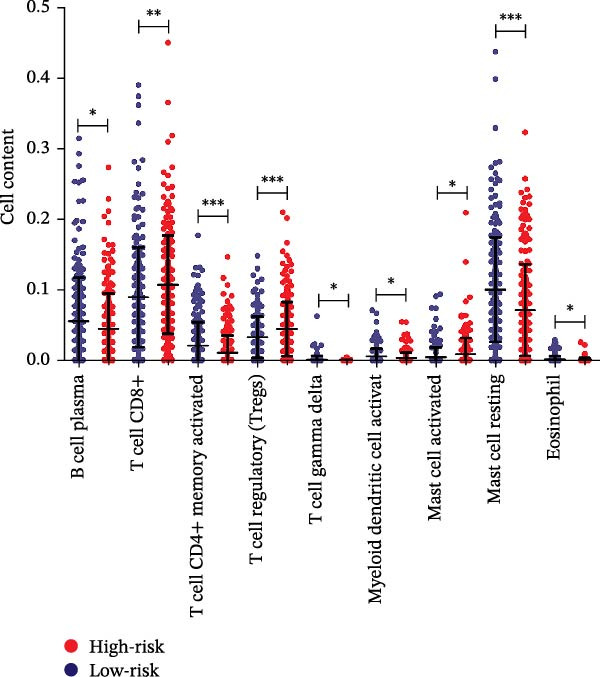
(A)

**Figure:**
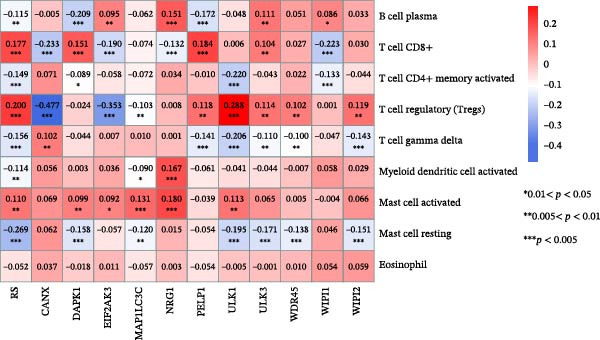
(B)

### 3.7. Validation of Optimal Prognostic Signature DAGs

**Figure 9:**
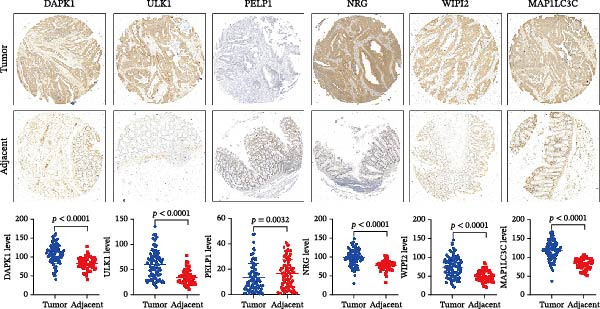
Validation of optimal prognostic signature DAGs expression in CRC and adjacent tissues. An immunohistochemistry assay was utilized to analyze the protein expression of ULK1, WIPI2, NRG1, PELP1, DAPK1, and MAP1LC3C ULK1, WIPI2, NRG1, PELP1, DAPK1, and MAP1LC3C.

Subsequently, we carefully chose a set of promising prognostic signature DAGs for detailed examination. Our method involved assessing their protein expression levels in CRC tissues through a tissue microarray analysis. As shown in Figure 9, this analysis revealed a significant increase in the expression of proteins such as DAPK1, ULK1, NRG, WIPI2, and MAP1LC3C in CRC tissues compared to their levels in adjacent noncancerous tissues. Conversely, PELP1 exhibited a decreased expression level in CRC tissues. A notable aspect of our study was the comparison with prior bioinformatics analysis, which showed a largely similar trend in the expression of these prognostic signature DAGs. However, there were notable exceptions: DAPK1 and MAP1LC3C, contrary to the tissue microarray findings, were indicated to be downregulated in CRC in the bioinformatics analysis, presenting a nuanced view of their behavior in cancer pathology.

### 3.8. Functional Validation of WIPI2 in CRC Cells

Based on tissue microarray analysis, several prognostic signature DAGs exhibited differential expression in CRC tissues, among which WIPI2 was markedly upregulated. Given its high expression, WIPI2 was selected for further functional investigation. SW480 cells, which displayed the highest WIPI2 levels among the tested CRC cell lines (Figure 10A), were used for knockdown experiments. SW480 cells were transfected with three WIPI2‐specific siRNAs (si‐WIPI2#1–#3) or si‐NC. Knockdown efficiency was evaluated by quantitative real‐time PCR and Western blot (Figure 10B,C), with si‐WIPI2#3 achieving the most effective suppression and subsequently used for all functional assays. Functional analyses demonstrated that WIPI2 knockdown significantly inhibited cell proliferation, as evidenced by decreased cell viability in the CCK‐8 assay (Figure 10D) and reduced EdU incorporation (Figure 10E). Autophagic flux was also impaired following WIPI2 depletion, with tandem mCherry–GFP–LC3B reporter analysis showing fewer red‐only puncta, indicative of suppressed autophagosome maturation (Figure 10F). Consistently, Western blot revealed decreased LC3B‐II and increased p62 levels (Figure 10G). Moreover, WIPI2 silencing promoted apoptosis, as indicated by elevated cleaved caspase‐3 expression (Figure 10G).

Figure 10Functional validation of WIPI2 in CRC cells. (A) Western blot analysis of WIPI2 expression in FHC, HT29, SW480, and HCT116 cells. SW480 cells exhibited the highest WIPI2 levels. (B–C) Quantitative real‐time PCR (B) and Western blot (C) verification of WIPI2 knockdown efficiency in SW480 cells transfected with si‐WIPI2#1, si‐WIPI2#2, si‐WIPI2#3, or si‐NC. si‐WIPI2#3 showed the most effective knockdown. (D) CCK‐8 assay showing reduced cell viability in WIPI2‐silenced SW480 cells. (E) EdU incorporation assay indicating decreased proliferation following WIPI2 knockdown. Scale bar = 20 μm. (F) Tandem mCherry–GFP–LC3B reporter assay demonstrating impaired autophagic flux in si‐WIPI2#3 cells. Yellow puncta (GFP^+^/mCherry^+^) represent autophagosomes, and red‐only puncta (GFP^−^/mCherry^+^) represent autolysosomes. Scale bar = 20 μm. (G) Western blot analysis of LC3B, p62, and cleaved caspase‐3 in SW480 cells following WIPI2 knockdown. GAPDH was used as a loading control. Data are presented as mean ± SD (*n* = 3).  ^∗∗∗^ *p* < 0.001, compared with si‐NC.

**Figure:**
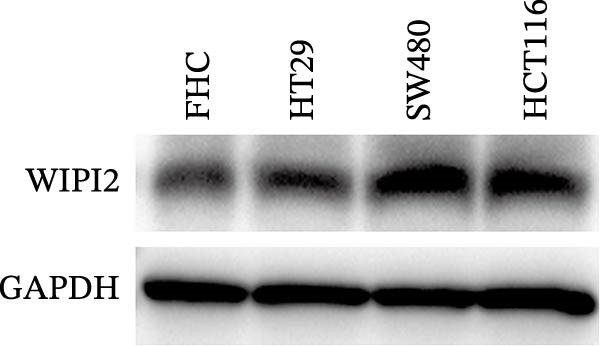
(A)

**Figure:**
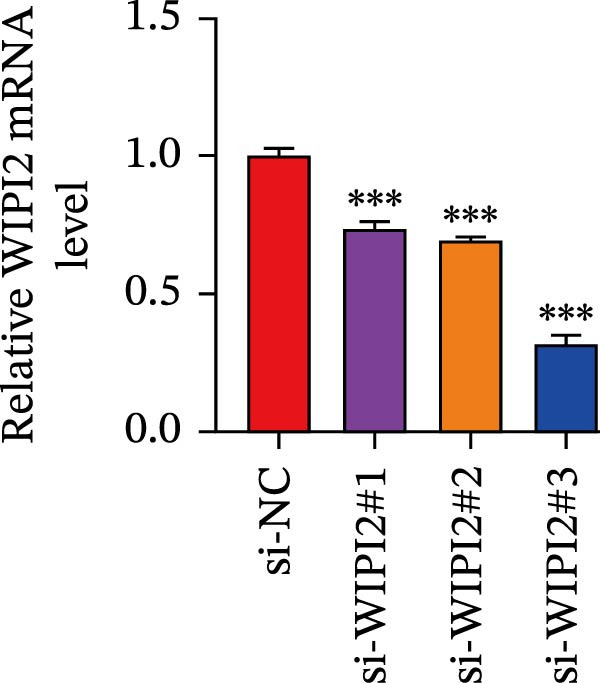
(B)

**Figure:**
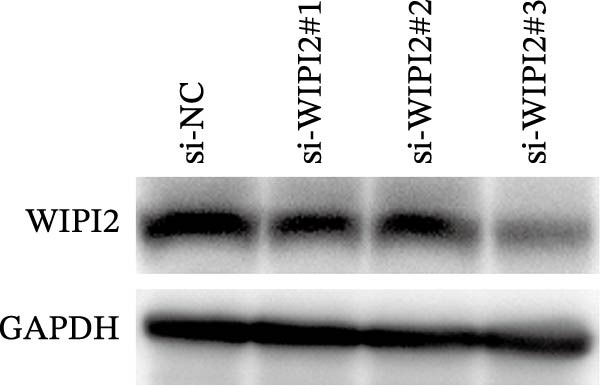
(C)

**Figure:**
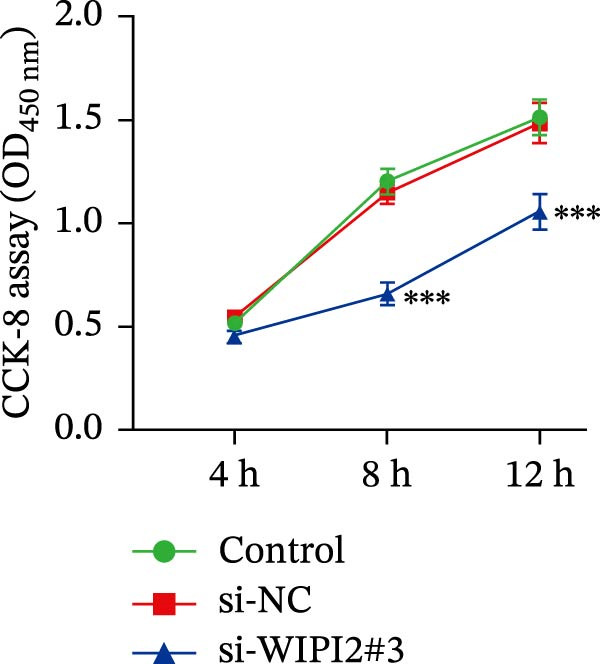
(D)

**Figure:**
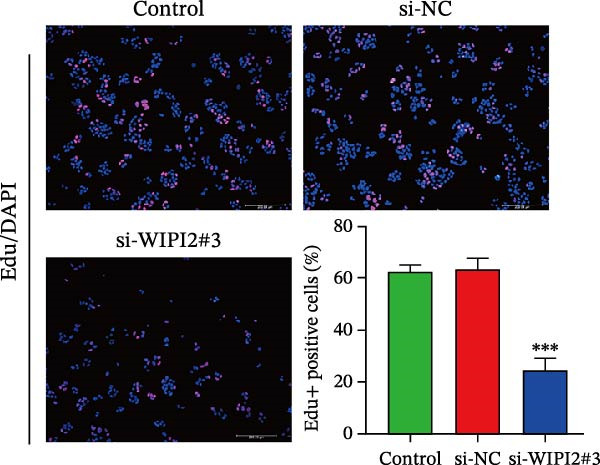
(E)

**Figure:**
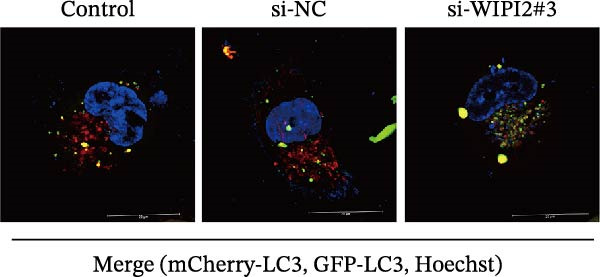
(F)

**Figure:**
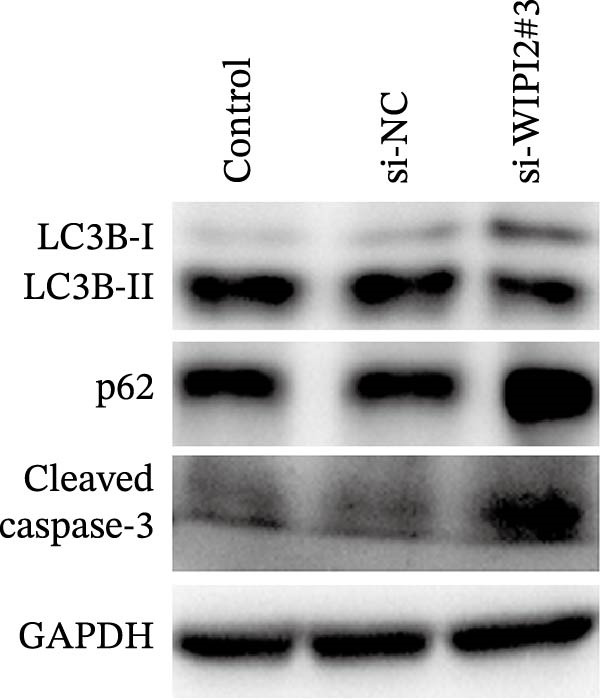
(G)

## 4. Discussion

Autophagy, an integral process of cellular self‐consumption, plays a contradictory role in tumor genesis and dissemination [23]. It serves as a crucial monitoring system that oversees the transformation of normal cells into cancerous ones. Concurrently, autophagy helps in averting cell death, supplying necessary nutrients, and cultivating drug resistance, thereby facilitating tumor initiation and development [24]. The complex molecular interplay of autophagy is recognized for its significant role in modulating chemoresistance in CRC, viewed through clinical, biological, and mechanistic lenses [25]. Therefore, developing a comprehensive autophagy‐related signature is critical for a deeper understanding and effective management of CRC’s malignant progression.

In this research, we pinpointed 11 key prognostic signature DAGs (CANX, NRG1, WIPI1, EIF2AK3, WDR45, PELP1, ULK1, WIPI2, DAPK1, ULK3, and MAP1LC3C) and developed a prognostic signature centered on these genes for autophagy‐related processes. External validation of our findings indicates that this signature robustly and independently stratifies the risk for CRC patients. Among these genes, CANX (calnexin) is notable as a critical regulator of the leucine‐stimulated mechanistic target of rapamycin kinase complex 1 [26], and it has been observed to significantly enhance CD8+ T‐cell‐mediated immune responses in CRC both in vitro and in vivo [27]. WIPI1 and WIPI2 are key in autophagy, localizing to autophagic membranes [28, 29], with WIPI1 knockdown impacting virus‐induced autophagy, highlighting its role in the phagophore formation process [30]. EIF2AK3, also known as PERK, is integral in managing intracellular proteostasis through unfolded protein response and integrated stress response [31] and is recognized as an important gene linked to lung cancer risk due to its association with endoplasmic reticulum (ER) stress [32]. WDR45, a mammalian homolog of yeast Atg18, is crucial in autophagosome formation [33]. While these studies underscore the importance of these DAGs in autophagy, their specific biological functions in CRC warrant further exploration.

The intricate tumor microenvironment (TME) of CRC significantly impacts patient prognoses, which encompasses not only the tumor cells but also includes tumor‐associated fibroblasts, endothelial cells, and various infiltrating immune cells [34]. Recognizing the critical role of immune cell infiltration in solid tumors, our study delved into the influence of prognostic signature DAGs on the infiltration levels of diverse immune cells. We noted a higher infiltration ratio of T cells CD8+, regulatory T cells (Tregs), and mast cells in the autophagy high‐risk group. Conversely, there was a greater abundance of B cell plasma, activated memory T cell CD4+, T cell gamma delta, activated myeloid dendritic cells, resting mast cells, and eosinophils in the autophagy low‐risk group. Previous studies have linked increased infiltration by cytotoxic T cells, memory T cells, and T helper cells with improved survival prospects [35]. Antigen‐specific B and T lymphocytes collaboratively orchestrate the immune response against tumor growth and progression [36].Additionally, pancreatic cancer research indicates that patients with a higher percentage of myeloid dendritic cells in cancer tissue have a longer survival compared to those with lower percentages or numbers in the peripheral blood [37].Thus, our analysis of TIICs suggests that a potent immunosuppressive microenvironment, marked by immune checkpoint inhibitors, in high‐risk patients might result in a diminished response to immunotherapies.

Importantly, we focused on validating the expression levels of six pivotal prognostic signature DAGs (NRG1, PELP1, ULK1, WIPI2, DAPK1, and MAP1LC3C). To our knowledge, death‐associated protein kinase (DAPK1) has been identified as a facilitator of gastric cancer cell migration and invasion, leading to the establishment of four DAPK1‐related signature genes that independently predict survival in gastric cancer patients [38]. In prostate cancer, however, DAPK1 functions as a tumor suppressor, attributed to its roles in inhibiting cellular transformation and metastasis [39]. A recent study by Wang et al. [40] corroborated this by demonstrating DAPK1’s downregulation in colon cancer tissues through immunohistochemistry. This variability in DAPK1 expression can be attributed to differences in tumor types, tissue sources, and even subtypes within the same tumor. Regarding MAP1LC3C (LC3C), a vital protein in autophagosome formation, it is one of the two paralogs, LC3B and LC3C, with contrasting activities in renal cancer [41]. Given the dual nature of autophagy in organisms, it’s understandable that MAP1LC3C expression may vary in tumor tissues. Importantly, among six prognostic signature DAGs, WIPI2 was highly expressed in SW480 CRC cells and selected for functional validation. Knockdown of WIPI2 inhibited proliferation, suppressed autophagy (decreased LC3B‐II, increased p62), and promoted apoptosis (increased cleaved caspase‐3), highlighting its key role in CRC cell survival and autophagic regulation.

Building on these findings, the autophagy‐related prognostic signature has potential for clinical application. It could be used to stratify CRC patients into high‐ and low‐risk groups using standard assays such as quantitative real‐time PCR or immunohistochemistry. High‐risk patients with elevated WIPI2 or other key DAGs may benefit from autophagy‐targeted therapies or tailored immunotherapy, as suggested by the observed correlations with immune infiltration. This approach provides a practical path for translating our molecular findings into clinical decision‐making.

## 5. Conclusion

To conclude, our research effectively pinpointed 11 crucial prognostic signature DAGs (CANX, NRG1, WIPI1, EIF2AK3, WDR45, PELP1, ULK1, WIPI2, DAPK1, ULK3, and MAP1LC3C) to formulate a novel autophagy‐related signature. This signature not only facilitates the prognostic assessment of CRC patients but also holds promise in differentiating responses to targeted therapies in specific cancer types. Nevertheless, the deeper molecular mechanisms underlying these findings warrant additional experimental exploration.

## Author Contributions

Dazhuang Miao, Yushui Song, and Jinxue Tong contributed to the study conception and design. Liang Zhou, Guanying Liang, Yan Wang, Wei He, Luyu Huang, Hongnan Lu, Shixiong Jiang, and Yunhe Jia collected the data and performed the data analysis. Wei He, Luyu Huang, Hongnan Lu, Shixiong Jiang, Dazhuang Miao, Liang Zhou, Zhiwei Li, and Jinxue Tong contributed to the interpretation of the data and the completion of figures and tables. Dazhuang Miao, Liang Zhou, and Jinxue Tong contributed to the drafting of the article and final approval of the submitted version.

## Funding

This work was supported by grants obtained from the Haiyan research Fund surface project (Grant JJMS2021‐04) and the Key Project of Haiyan Research Fund (Grant JJZD2018‐03).

## Disclosure

The authors should state that the paper is published in a preprint. A preprint has previously been published [42].

## Ethics Statement

Ethical approval was given by the Ethics Committee of Harbin Medical University Cancer Hospital (Number KY2021‐31).

## Conflicts of Interest

The authors declare no conflicts of interest.

## Supporting Information

Additional supporting information can be found online in the Supporting Information section.

## Supporting information


**Supporting Information 1** Figure S1: Heatmap of 170 differentially expressed autophagy‐related genes (DAGs) between CRC and normal tissues in the TCGA dataset. Figure S2: LASSO regression model. (A) The LASSO coefficient profiles for the 25 prognosis‐related DAGs and the cross‐validation were used to tune the parameter screening in the LASSO regression model. (B) The LASSO coefficient of 11 optimal prognostic signature DAGs. Figure S3: Expression levels of optimal prognostic signature DAGs. Left panel in TCGA (A) and GSE44076 (B): Depicts the expression levels of 11 genes in tumor and control samples. Right: Presents the ROC curve for sample type recognition based on the expression levels of 11 genes. The numbers in brackets indicate the specificity and sensitivity of the ROC curve.

**Supporting Information 2** Table S1: Total 170 differentially expressed autophagy‐related genes (DAGs) were identified and shown.

**Supporting Information 3** Table S2: Total 11 optimal prognostic signature DAGs were extracted and compared in the TCGA and GSE44076 datasets.

**Supporting Information 4** Table S3: The expression levels of 11 genes in high and low autophagy score groups were presented.

**Supporting Information 5** Table S4: Total 1320 significantly differentially expressed genes (SDGs) between the high‐risk and low‐risk groups were shown in TCGA CRC samples.

**Supporting Information 6** Table S5: These SDGs were significantly enriched in 31 GO biological process categories and 10 KEGG pathways.

**Supporting Information 7** Table S6: The correlation between the risk score (RS) and 22 types of TIICs in CRC was displayed.

## Data Availability

The data that support the findings of this study are available from the corresponding author upon reasonable request.
