# Supplementary material for: Analysis of Autophagy‐Related Gene Signature Associated With Clinical Prognosis and Immune Microenvironment in Colorectal Cancer
Source: Mediators Inflamm. 2026 Mar 28;2026:3900151. doi: 10.1155/mi/3900151 (PMC13140268; doi:10.1155/mi/3900151)
Supplement: Supplementary file 1 — Supporting Information 1 Figure S1: Heatmap of 170 differentially expressed autophagy‐related genes (DAGs) between CRC and normal tissues in the TCGA dataset. Figure S2: LASSO regression model. (A) The LASSO coefficient profiles for the 25 prognosis‐related DAGs and the cross‐validation were used to tune the parameter screening in the LASSO regression model. (B) The LASSO coefficient of 11 optimal prognostic signature DAGs. Figure S3: Expression levels of optimal prognostic signature DAGs. Left panel in TCGA (A) and GSE44076 (B): Depicts the expression levels of 11 genes in tumor and control samples. Right: Presents the ROC curve for sample type recognition based on the expression levels of 11 genes. The numbers in brackets indicate the specificity and sensitivity of the ROC curve. [file MI-2026-3900151-s001.docx]

**Supplement material**

**Figure S1** Heatmap of 170 differentially expressed autophagy-related genes (DAGs) between CRC and normal tissues in TCGA dataset.


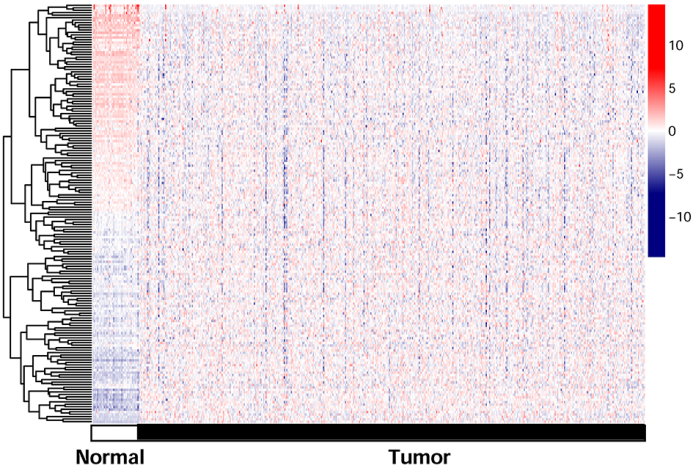


**Figure S2 LASSO regression model.** (A) The LASSO coefficient profiles for the 25 prognosis-related DAGs and Cross-validation was used to tune the parameter screening in the LASSO regression model. (B) The LASSO coefficient of 11 optimal prognostic signature DAGs.


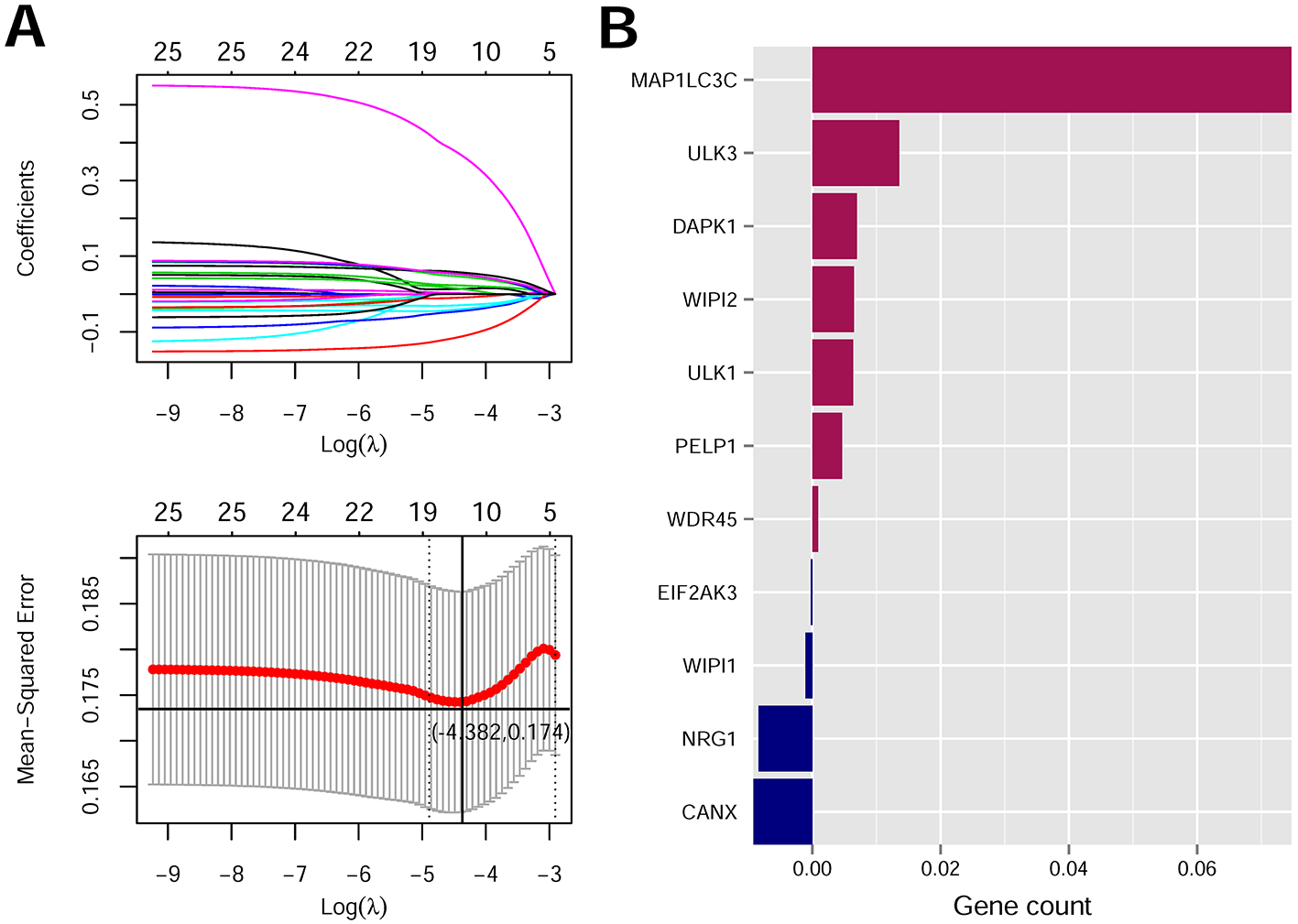


**Figure S3 Expression levels of optimal prognostic signature DAGs.** Left panel in TCGA (A) and GSE44076 (B): Depicts the expression levels of 11 genes in tumor and control samples. Right: Presents the ROC curve for sample type recognition based on the expression levels of 11 genes. The numbers in brackets indicate the specificity and sensitivity of the ROC curve.


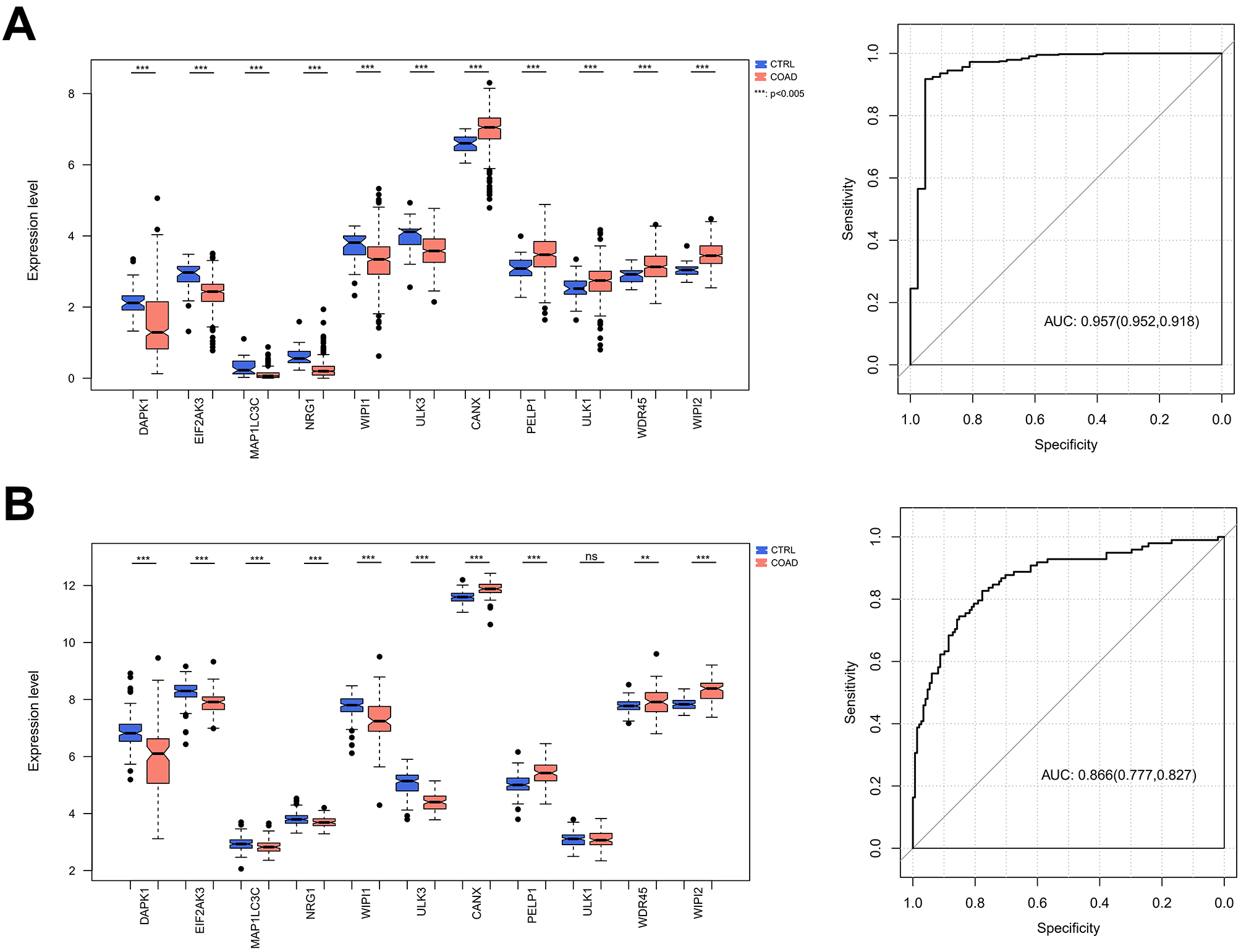


**Table S1** Total 170 differentially expressed autophagy-related genes (DAGs) were identified and shown.

**Table S2** Total 11 optimal prognostic signature DAGs were extracted and compared in the TCGA and GSE44076 datasets.

**Table S3** The expression levels of 11 genes in high and low autophagy score groups were presented.

**Table S4** Total 1320 significantly differentially expressed genes (SDGs) between the high-risk and low-risk groups were shown in TCGA CRC samples

**Table S5** These SDGs were significantly enriched in 31 GO biological process categories and 10 KEGG pathways.

**Table S6** The correlation between the risk-score (RS) and 22 types of TIICs in CRC were displayed.
